# How are newborns fed in their first day of life in Morocco? a survey

**DOI:** 10.11604/pamj.2020.37.375.25250

**Published:** 2020-12-23

**Authors:** Nour Mekaoui, Fatima Zahra Belkouchi, Idriss Chajai, Badr Sououd Benjelloun Dakhama, Lamya Kerboubi

**Affiliations:** 1 Laboratory of Biostatistics, Clinical Research and Epidemiology, Faculty of Medicine and Pharmacy, Mohammed V University of Rabat, Rabat, Morocco,; 2 Department of Gynecology, Obstetrics, Oncology and High-Risk Pregnancy, Souissi Maternity Hospital Rabat, Rabat, Morocco,; 3 Pediatric Medical Emergency Department, Rabat´s Children´s Hospital Rabat, Rabat, Morocco

**Keywords:** Breastfeeding, newborn, Morocco

## To the editors of the Pan African Medical Journal

Breastfeeding is recommended as the best feeding for a newborn. Benefits on both the mother and the newborn health has been clearly established [1]. We conducted a study which aims to evaluate the mother's knowledge and practices of newborns feeding. This cross-sectional study was carried out between the 1^st^ of January 2019 and the 31^st^ of December 2019. It included 1000 mother newborn couples who consulted Rabat´s pediatric emergencies department during the study period. They had to fill out an anonymous survey, and be discharged at the end of the consultation without transfer to the neonatology department in order to be included in our study. This survey allowed collecting data, concerning: socio economic and demographic profile of mothers; characteristic of pregnancy and childbirth; feeding patterns and their reasons; mother's sources of information. Thus, mean age of the mothers was 29 years old. Lowest and highest extremes were 18 and 46 years old respectively. One percent (1%) of cases were single mothers. 78% of mothers were housewives, while parent's socioeconomic status was low in 48% of cases, with 270 illiterate mothers. Seventy-six percent (76%) of cases came from urban districts. We found that pregnancy was desired in 90% of cases and followed in 80% of cases. There was a vagina delivery in 78% of cases, with full term delivery in 94% of cases, and in a medicalized environment in 86% of cases. Prevalence of breastfeeding in our study is 78%, with a first feeding in 45% cases in the first three hours. Thirty-five percent (35%) were first fed between 3 and 6 hours, and 20% after the 6^th^ hour. Artificial breastfeeding was retrieved in 31% of cases, and the main reasons justifying that behavior were the absence of milky rise or maternal fatigue.

Fifty-five percent (55%) of the babies received a nondairy food (Table 1). In these cases, the reason invocated was preventing infant colic. At last, 35% of mothers were sensitized and benefited from prenatal information on breastfeeding. Our study remains the first to focus on the attitudes and practices of the mothers in feeding the newborn during the first day of life on national level. These mothers display disparate socioeconomic, demographic and intellectual levels allowing us to survey a representative specimen, and study the influence of all these factors on the newborn feeding. We remind that available studies about breastfeeding in Morocco are a wake up call to the decline observed in the practice of breastfeeding. Indeed, a comparison with a 2006 study conducted at Souissi maternity hospital in Rabat shows a decline of the prevalence of breastfeeding, from 91% [2] to 78% in 2019. In an international scale, we have strong evidence from Canada showing a greater prevalence of breastfeeding in the first days of life with 90% [3]. Prevalence in France is close to ours at 74% [4], while prevalence in Iran remains low at 53% [5]. Only 35% of mothers are sensitized while 80% of pregnancies are followed, which shows a lack of sensitization. Despite that, the prevalence remains high at 78%. We remind that the Moroccan Ministry of Health has set up since 1991 a national plan of action to promote breastfeeding. In order to help and encourage young mothers to continue breastfeeding when they leave the maternity ward, professionals are advised to provide support and information strategies. It is also the recommendations of the High Authority for Health (HAS) for the maternal breastfeeding support process released in 2006. At last, there is a shift from rural to urban districts.

**Table 1:** characteristic of newborn's feeding in their first day of life in Morocco

Variables	Effective (%)
**Breastfeeding**	
Yes	780 (78)
No	220 (22)
**First breastfeed**	
Before H3	450 (45)
Between H3 and H6	350 (35)
After H6	200 (20)
**Artificial breastfeeding**	
Yes	690 (69)
No	310 (31)
**Non-dairy foods**	
Yes	550 (55)
No	450 (45)

These young mothers are often exposed to aggressive marketing and environment influence, both promoting feeding with “milk formula”, while mothers in rural districts tend to breastfeed. This study allows us to observe that there is a decline in breastfeeding in Morocco, and lot of gaps regarding feeding patterns and behaviors. To improve infant feeding practices, it would be important to raise awareness among health professionals on this topic.
